# Transplant Trial Watch

**DOI:** 10.3389/ti.2023.11045

**Published:** 2023-01-13

**Authors:** John M. O’Callaghan

**Affiliations:** ^1^ University Hospitals Coventry and Warwickshire, Coventry, United Kingdom; ^2^ Centre for Evidence in Transplantation, University of Oxford, Oxford, United Kingdom

**Keywords:** kidney transplantation, immunosuppression, systematic review, liver transplantation, octreotide

To keep the transplantation community informed about recently published level 1 evidence in organ transplantation ESOT and the Centre for Evidence in Transplantation have developed the Transplant Trial Watch. The Transplant Trial Watch is a monthly overview of 10 new randomised controlled trials (RCTs) and systematic reviews. This page of Transplant International offers commentaries on methodological issues and clinical implications on two articles of particular interest from the CET Transplant Trial Watch monthly selection. For all high quality evidence in solid organ transplantation, visit the Transplant Library: www.transplantlibrary.com.

Randomised Controlled Trial 1 Revisiting maintenance immunosuppression in patients with renal transplant failure: early weaning of immunosuppression versus prolonged maintenance-systematic review and meta-analysis.

*by Elgenidy, A., et al. Journal of Nephrology [Online ahead of print].*

## Aims

This study aimed to evaluate whether early or late withdrawal of maintenance immunosuppression in patients with kidney transplant failure is linked with better outcomes.

## Interventions

Electronic databases including PubMed, WOS, Ovid, and Scopus databases were searched. Titles and abstracts were screened for eligiblity by four independent reviewers. Data extraction was conducted by two independent reviewers. The Newcastle–Ottawa Scale was used to assess the methodological quality of the included studies.

## Participants

10 studies were included in the review.

## Outcomes

The outcomes of interest were incidence of infection, cancer, mortality (infection-related, malignancy-related, cardiovascular-related), transplant nephrectomy, re-transplantation, panel reactive antibody (PRA) and admission to hospital.

## Follow-Up

Not applicable.

## CET Conclusion

This systematic review and meta-analysis investigated the role of continued immunosuppression in the patient with a failed kidney transplant. The authors identified 10 studies including 1,187 patients. There was no difference in overall survival, sensitisation, malignancy, infection or retransplant rates between patients who withdrew immunosuppression early or remained on maintenance immunosuppression. Review methodology appears good, with independent reference screening and searches across multiple databases. The majority of studies were retrospective cohorts with a risk of selection bias. Many meta-analyses included only 2 or 3 studies, with quite wide confidence intervals around estimated effect sizes and some degree of heterogeneity. It is possible, therefore, that a true effect could have been missed due to selection bias or lack of power.

## Trial Registration

Not applicable.

## Funding Source

Not reported.

Randomised Controlled Trial 2 Impact of Octreotide on Early Complications After Liver Transplant: A Randomized, Double-Blind Placebo-Controlled Trial.

*by Bagheri Lankarani, K., et al. Experimental & Clinical Transplantation 2022; 20(9):835–841.*

## Aims

This study aimed at investigating the role of octreotide on early outcomes following liver transplantation.

## Interventions

Participants were randomised to receive either octreotide or placebo.

## Participants

50 patients who underwent deceased donor orthotopic liver transplantation.

## Outcomes

The primary endpoint was renal function. The secondary endpoints were length of intensive care unit (ICU) and hospital stays, rate of nosocomial infection, and rate of early allograft dysfunction (EAD).

## Follow-Up

16.4 days posttransplant.

## CET Conclusion

This is a clear report of a randomised controlled study in liver transplantation. A prior power calculation was conducted, and the trial was therefore adequately powered. The method of randomisation was through allocation software. The trial is described as double-blinded; however, the trial drugs were provided in vials labelled as either “A” or “B.” This is unfortunately not as robust for maintaining blinding as having individual codes for every dose administered. The study found a significant reduction in AKI after liver transplantation when octreotide was administered. This was particularly for stage II AKI. As the diagnosis of AKI includes drop in urine output, this is not as robust as relying on serum markers alone. There was also a significant reduction in early allograft dysfunction with octreotide (16% versus 47%). The length of ICU stays, and total hospital stay were also reduced. It is speculated that all these effects are a result of improved renal perfusion and the anti-inflammatory effect of octreotide. A reduction in nosocomial infection was also seen with octreotide, but the mechanism of action for this is not clear.

## Jadad Score

5.

## Data Analysis

Strict intention-to-treat analysis.

## Allocation Concealment

Yes.

## Trial Registration

IRCT20190619043942N1.

## Funding Source

Non-industry funded.

## Clinical Impact Summary

This is an interesting RCT in liver transplantation investigating the potential for octreotide infusion to reduce the risk of post-operative Acute Kidney Injury (AKI). The study was relatively small, with only 50 transplants included across both arms. However, the scale of the reduction in AKI was so great that a significant reduction was demonstrated with octreotide; The study group had a 20% risk of AKI compared to 44% in the control group. The difference was particularly evident in stage 2 AKI. There was also a significant reduction in early allograft dysfunction of a similar magnitude (16% versus 47%). A significant reduction in post-operative infections was also seen with octreotide, but the mechanism for this is not clear.

There are some concerns about the methodology that do weaken the strength of the conclusions though. For example, the use of vials marked as A or B can lead to the loss of allocation concealment, despite an otherwise acceptable method of randomisation.

Octreotide has some evidence base in ischaemia reperfusion injury for both liver and kidney, that may support the results seen in this study. However, the scale of the apparent benefit was really quite extreme in this study. As the study authors acknowledge, further studies are needed to really understand if this is a true effect of the intervention alone.

